# Genetical Genomics Reveals Large Scale Genotype-By-Environment Interactions in *Arabidopsis thaliana*

**DOI:** 10.3389/fgene.2012.00317

**Published:** 2013-01-10

**Authors:** L. Basten Snoek, Inez R. Terpstra, René Dekter, Guido Van den Ackerveken, Anton J. M. Peeters

**Affiliations:** ^1^Laboratory of Plant Ecophysiology, Department of Biology, Institute of Environmental Biology, Utrecht UniversityUtrecht, Netherlands; ^2^Laboratory of Plant-Microbe Interactions, Department of Biology, Institute of Environmental Biology, Utrecht UniversityUtrecht, Netherlands; ^3^Centre for BioSystems Genomics, Wageningen University and Research CenterWageningen, Netherlands

**Keywords:** genetical genomics, genotype-by-environment interaction, *Arabidopsis thaliana*, shade avoidance, expression-QTLs, recombinant inbred lines

## Abstract

One of the major goals of quantitative genetics is to unravel the complex interactions between molecular genetic factors and the environment. The effects of these genotype-by-environment interactions also affect and cause variation in gene expression. The regulatory loci responsible for this variation can be found by genetical genomics that involves the mapping of quantitative trait loci (QTLs) for gene expression traits also called expression-QTL (eQTLs). Most genetical genomics experiments published so far, are performed in a single environment and hence do not allow investigation of the role of genotype-by-environment interactions. Furthermore, most studies have been done in a steady state environment leading to acclimated expression patterns. However a response to the environment or change therein can be highly plastic and possibly lead to more and larger differences between genotypes. Here we present a genetical genomics study on 120 *Arabidopsis thaliana*, Landsberg *erecta* × Cape Verde Islands, recombinant inbred lines (RILs) in active response to the environment by treating them with 3 h of shade. The results of this experiment are compared to a previous study on seedlings of the same RILs from a steady state environment. The combination of two highly different conditions but exactly the same RILs with a fixed genetic variation showed the large role of genotype-by-environment interactions on gene expression levels. We found environment-dependent hotspots of transcript regulation. The major hotspot was confirmed by the expression profile of a near isogenic line. Our combined analysis leads us to propose CSN5A, a COP9 signalosome component, as a candidate regulator for the gene expression response to shade.

## Introduction

Developmental processes and the responses of organisms to their environment are largely genetically determined. However, phenotypic variation is also strongly influenced by the environment and by genotype-by-environment interactions. Complex interactions between polymorphic gene products specific for an environment or changing conditions are at the basis of the variation in responses between individuals within a species. Besides phenotypic responses, transcript levels of many genes are similarly influenced by the environment in addition to genetic differences.

Variation in genotype leading to heritable differences in transcript levels has been used to find expression Quantitative Trait Loci (eQTLs) in genetical genomics studies (Jansen and Nap, 2001) on a number of model organisms (Brem et al., 2002; Bing and Hoeschele, 2005; Brem and Kruglyak, 2005; Bystrykh et al., 2005; Li et al., 2006, 2010; Keurentjes et al., 2007; West et al., 2007; Viñuela et al., 2010, 2012; Zhang et al., 2011). The identified eQTLs were instrumental in unraveling transcript regulatory networks (Bing and Hoeschele, 2005; Keurentjes et al., 2007; Terpstra et al., 2010). DNA polymorphisms in or near a gene can affect the gene’s own expression (local or *cis* regulation) and/or the expression of target genes at other locations in the genome (distant or *trans*-regulation). Candidate regulatory genes, responsible for distant regulation, can be selected based on co-expression with their target genes. Regulatory loci that affect the expression of many genes are called Hotspots for transcript regulation (HTRs). These genomic regions, enriched in eQTLs, are identified in most genetical genomics experiments (Brem et al., 2002; Schadt et al., 2003; Bystrykh et al., 2005; Li et al., 2006; Keurentjes et al., 2007; West et al., 2007; Zhang et al., 2011; Snoek et al., 2012) and point to, so called, master regulators. Regulatory genes do not always exert their effect on target gene expression by changing their own expression levels, but instead by differential activity at the protein level. In our previous genetical genomics study in *Arabidopsis thaliana* (Keurentjes et al., 2007) the receptor-like kinase (RLK) gene *ERECTA* was identified as an HTR, although it was not *cis-*regulated. The combination of single mutant gene expression profiles with eQTL data and transcription factor binding site analysis, allowed the verification of HTR targets and identification of downstream signaling cascades (Terpstra et al., 2010).

Genetical genomics studies on *Arabidopsis* performed so far have revealed variation in gene expression under one experimental condition (Keurentjes et al., 2007; West et al., 2007; Zhang et al., 2011). Steady state transcript levels were measured and the effect of genotype within these environmental settings on variation in gene expression was studied. The genotype of an organism can in fact express different phenotypes as a function of the environment, which is called phenotypic plasticity (Nicotra et al., 2010). Many of these plastic responses to a change in the environment are relatively fast and the result of transient changes in transcript abundance. Plasticity in gene expression is much exploited in numerous gene expression analyses studying the effect of specific treatments. When different genotypes respond differently to changes in the environment, there is genotype-by-environment interaction. In any case though, this interaction will only manifest itself after induction, for instance after an environmental change.

The influence of the environment on gene expression is reflected in the highly plastic nature of gene expression regulation, as shown in a genetical genomics studies on a *Caenorhabditis elegans* recombinant inbred line (RIL) population grown in two different temperatures (Li et al., 2006) or at three different ages (Viñuela et al., 2010). The majority of genes that showed plasticity in regulation due to genotype-by-temperature interaction were mainly regulated in *trans*, with a large group of genes affected by the same regulatory locus. Also in yeast, grown on two different carbon sources, *trans-*regulation was more plastic (Smith and Kruglyak, 2008). In another study, where yeast strains were grown under distinct growth conditions, the genes that showed plastic regulation were biased to non-essential genes (Landry et al., 2006). In contrast, analysis of dynamic responses to heat-shock revealed that plasticity in regulation was more often observed for essential genes (Eng et al., 2010). The latter study clearly showed that, in addition to different environments, the transition between environments provided a new source of expression variation.

An important environmental change that plants experience everyday is shade or a change in light conditions. Low light treatment induces the shade avoidance syndrome (SAS), which includes enhanced elongation of stems and petioles, upward leaf movement (hyponastic growth) and increased apical dominance (Ballaré, 1999; Millenaar et al., 2005; Franklin, 2008; Keuskamp et al., 2011), concurrent with rapid changes in gene transcript levels (Devlin et al., 2003; Salter et al., 2003; Sessa et al., 2005; Millenaar et al., 2006). Low light is perceived by phytochromes, cryptochromes, phototropins, and members of the Zeitlupe (ZTL) family of photoreceptors (Takemiya et al., 2005; Franklin, 2008; Demarsy and Fankhauser, 2009; Pierik et al., 2009; Keller et al., 2011). Subsequent signaling involves phytochrome-interacting factors PIF4 and PIF5. Although the low light-induced hyponastic response is similar to ethylene-induced hyponasty, the low light response is independent of ethylene signaling. Instead, auxin signaling and polar transport in combination with brassinosteroids (BR) are required for low light-induced hyponastic growth (Millenaar et al., 2009; Kozuka et al., 2010; Keller et al., 2011; Keuskamp et al., 2011).

Here we analyze the effect of genotype and the genotype-by-environment interaction on genome wide transcript levels of *Arabidopsis* RILs exposed to low light conditions. Transcript levels of 120 Landsberg *erecta* (L*er*) and Cape Verde Islands (Cvi) RILs (Alonso-Blanco et al., 1998) were measured on DNA microarrays and compared to our previous genetical genomics experiment (GG1) on the same population that was grown under normal light conditions (Keurentjes et al., 2007). Comparison of the two experiments identified genotype-specific eQTLs as well as experiment-specific, so called plasticity eQTLs. In response to low light a major effect on transcript regulation was exerted by an HTR on chromosome 1, which was confirmed in the analysis of a near isogenic line (NIL). Genes that are regulated by the HTR were associated with the circadian clock and processes related to auxin, sterols and reactive oxygen species (ROS). We propose a candidate master regulator to interactively affect these processes in the complex low light-induced SAS.

## Results

### Natural variation in transcript abundance under low light conditions

The RILs generated from the parental *Arabidopsis* accessions L*er* and Cvi show a high level of phenotypic variation in response to low light, indicating there is a strong influence of genotype on the SAS-related responses (Van Zanten et al., 2010). To assess the effect of genotype on the transcriptional response to low light, genome wide transcript levels were measured in 120 L*er*/Cvi RILs and their parental lines (referred to as Genetical Genomics experiment 2, or GG2). For this, 3-week-old plants grown under short-day conditions were shifted to low light conditions, a ∼90% reduction in light intensity, and leaf tissue was collected 3 h later. We assessed the contribution of plant genotype to variation in transcript levels by calculating the broad sense heritabilities for all genes in the parental lines and RILs. The median broad sense heritability of gene expression in the parents was 0.26 and in the RILs 0.46, indicating that the genetic background has a greater influence on the variation in transcript levels in the RILs than in the parents. We determined linkage to variation in transcript abundance and mapped 7868 eQTLs for 6676 genes (at false discovery rate (fdr) < 0.05) for ∼32% of total measured transcripts.

**Figure 1:**
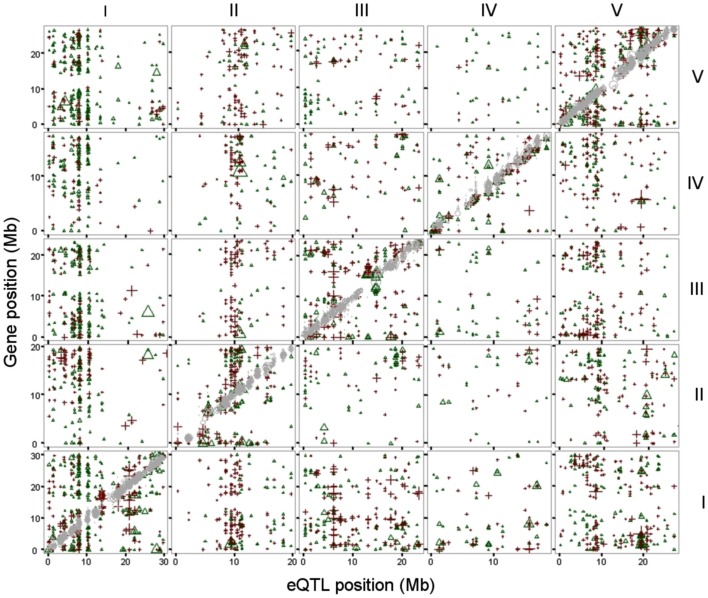
**eQTLs, physical position of the top of the eQTL (*x*-axis) plotted against the physical position of the gene (*y* axis)**. Both physical positions are in mega basepairs (Mb) and plotted per chromosome (I-V, indicated at the top and right side). eQTLs for local (cis) regulation are in gray (diagonal). eQTLs with a L*er* positive effect are depicted as green triangles, eQTLs with a Cvi positive (=L*er* negative) effect as red plus signs. Chromosomes are indicated on the right for rows and on top for columns of graphs.

This linkage was visualized by plotting the position of the genes of which expression levels are regulated to the position of the regulatory eQTLs (Figure 1). The gray diagonal line depicts local regulation. Distant regulation is visible as vertical “bands.” Many distant effects are found near the top of chromosome 1, where transcript levels of a large number of genes are affected by one locus. We found a similar number of eQTLs for distant as for local regulation (Table 1).

**Table 1:** **eQTLs mapped in 120 RILs exposed to low light conditions**.

Feature	Number	Fraction (%)
eQTL	7868	
Distant eQTL	4170	53.0
Genes with eQTL	6676	
Genes with local and distant eQTL	634	9.5
Genes with only local eQTL	3054	45.7
Genes with only distant eQTL	2988	44.8

The transcript profiles of L*er* and Cvi, similarly treated for 3 h with a ∼90% reduction in light intensity, showed 3228 genes (fdr < 0.05) with differentially abundant transcripts between the parental lines (Table 2). Of these genes, 2379 (74%) had an eQTL, which is 36%of all genes with an eQTL. The eQTLs for the L*er*/Cvi differentially abundant transcripts show more local regulation than the overall fraction of locally regulated genes (707 distant, 1374 local, and 298 both). These local eQTLs are probably caused by polymorphisms between L*er* and Cvi within the promoters or other regulatory sequences of the genes that show differential expression. To assess this relation, the eQTL distribution was correlated to the distribution of genes containing Single Nucleotide Polymorphisms (SNPs). Indeed, the local eQTL distribution shows a higher correlation to the SNP distribution (0.64; *t*-test *p* < 10^−14^) than that of the distant eQTLs (0.26; *t*-test *p* < 0.01).

**Table 2:** **eQTLs of L*er*/Cvi differentially abundant transcripts (DATs) in response to low light treatment**.

Feature	Number	Fraction (%)
DATs	3228	
DATs with eQTL	2379	73.7
DATs with local and distant eQTL	298	12.5
DATs with only local eQTL	1374	57.8
DATs with only distant eQTL	707	29.7

### Variation in gene expression increased by transgression and low light treatment

The number of genes with an eQTL, as determined in the RIL population, is much larger than the number of transcripts that are differential between L*er* and Cvi. For 64% of the genes with an eQTL (∼20% of all genes) no differential transcript abundance between the parental lines is observed. The influence of recombination of parental alleles in the RILs on the larger variation in transcript levels is reflected in the higher heritabilities for the transcript levels in the RILs than in the parents. Combinations of parental alleles with different effects can enhance variation and lead to more extreme transcript levels than in the parental lines. This transgression was observed for 3495 genes (17% of the genes). The genes with eQTLs but without differential transcript levels between the parents show more transgression (21%; 913 of 4297) than the overall fraction of transgressive genes with eQTLs (18%; 1230 of 6676 genes; hypergeometric test *p* < 1.3* 10^−16^). Transgression can therefore explain, for a considerable part, the larger variation in gene transcript levels caused by genotype in the RILs than in the parents.

A major part of the environmental effect in GG2 may well be attributed to the change in light conditions, the exposure to low light, which induces the SAS. To estimate the effect of natural variation within the RILs on the response induced by low light, we compared our data to transcript profiles of Columbia (Col-0) petioles treated with low light under similar experimental conditions (Millenaar et al., 2006). A reduction in light intensity of ∼90% for 3 h resulted in differential transcript levels of 2579 genes, compared to Col-0 plants that remained under full light conditions. Of these, 2278 low light responsive genes could be analyzed on the microarray in GG2, and are referred to as low light-regulated genes from here on. The number of low light-regulated genes that is differentially regulated between L*er* and Cvi is 506 (22%). This is significantly higher (hypergeometric test *p* < 1.8*10^−17^) than the overall percentage of 16% genes with differentially abundant transcripts (2794 genes of the 17498 genes that could be measured in both experiments). We also observe significantly more (hypergeometric test *p* < 1.3*10^−29^) eQTLs in GG2 for the low light-affected transcripts (43%) than for all transcripts (33%). Taken together this shows that the low light treatment of the RILs in GG2 revealed natural variation in the induced response to low light.

### Comparison of two genetical genomics experiments

**Figure 2:**
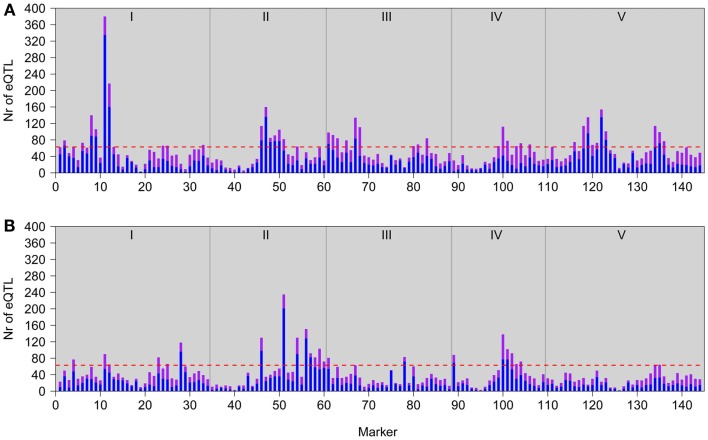
**Genomic distributions of eQTLs in GG2 (A; this study) and GG1 (B; Keurentjes et al., 2007)**. Bars represent the number of local (*in cis*; purple) and distant (*in trans*; blue) eQTL peaks detected at each marker position. The horizontal red lines represent the significance threshold values for defining a hotspot. Gray vertical lines depict chromosomal borders, chromosomes (I–V) are indicated at the top.

Our genetical genomics experiment under low light (GG2) gave us the opportunity to compare the observed natural variation in gene expression to our first genetical genomics experiment (GG1), under normal light conditions. In the two experiments the same population of RILs and parental lines were profiled in a similar microarray distant-pair design (Fu and Jansen, 2006). There were, however, marked differences. In GG1 the aerial parts of 7-day-old seedlings were profiled, opposed to leaves of 3-week-old plants in GG2. So not only developmental stage, but also the collected tissue types were different. Furthermore, seedlings in GG1 were grown *in vitro* on synthetic medium under long-day conditions. In GG2 the plants were grown under short-day conditions in soil. Most importantly, plants from GG2 were placed, 3 h before being harvested, to low light conditions, which mimics neutral shade that is known to lead to large transcriptional changes. In contrast, seedlings in GG1 were not subjected to low light but harvested for expression profiling when plants were 7 h into the light period. From here on we refer to these developmental and environmental differences as the experimental effect. In the two genetical genomics experiments the distributions of eQTLs over the five *Arabidopsis* chromosomes are clearly distinct, reflecting the strong experimental effect on the regulation of gene expression (Figure 2).

In GG2, the main HTR is located at the top of chromosome 1, where the L*er* locus mainly has a positive effect on gene expression (Figures 1 and 2A). This locus co-locates with a QTL for petiole angle, a phenotype that is part of the SAS (Snoek and Peeters, personal communication) and suggests a functional link between the expression changes caused by the chromosome 1 HTR and low light-induced phenotypic variation. In GG1, the gene underlying the main HTR (Figure 2B) was *ERECTA* on chromosome 2 that also has a large influence on many phenotypic growth-related traits (Van Zanten et al., 2009). Comparison of the eQTL distributions in both experiments shows a stronger correlation between GG1 and GG2 for local (∼0.64) than for distant eQTLs (∼0.21). This suggests that the eQTLs for local regulation are predominantly genotype-specific and independent of the experimental conditions, whereas those for distant regulation are more experimentally controlled.

In the two experiments together a total of 12287 eQTLs were mapped, for 7259 genes of the 19205 genes profiled in both experiments. Despite the completely different environmental conditions, for 1095 genes1129 overlapping eQTLs were found, that is 9% of the total number of eQTLs. As we expected, there was a strong effect of the genotype on the 1095 genes having overlapping eQTLs as 974 of these (89%) were found to be locally regulated, suggesting they are affected by *cis*-regulatory polymorphisms. The 11540 eQTLs that were specific for each experiment reflect the large plasticity in gene expression regulation. The fraction of distantly regulated eQTLs is considerably higher in the experiment-specific eQTLs (62%; 7158 eQTLs) than in the overlapping eQTLs (11%).

### Plastic regulation of transcript abundance

**Figure 3:**
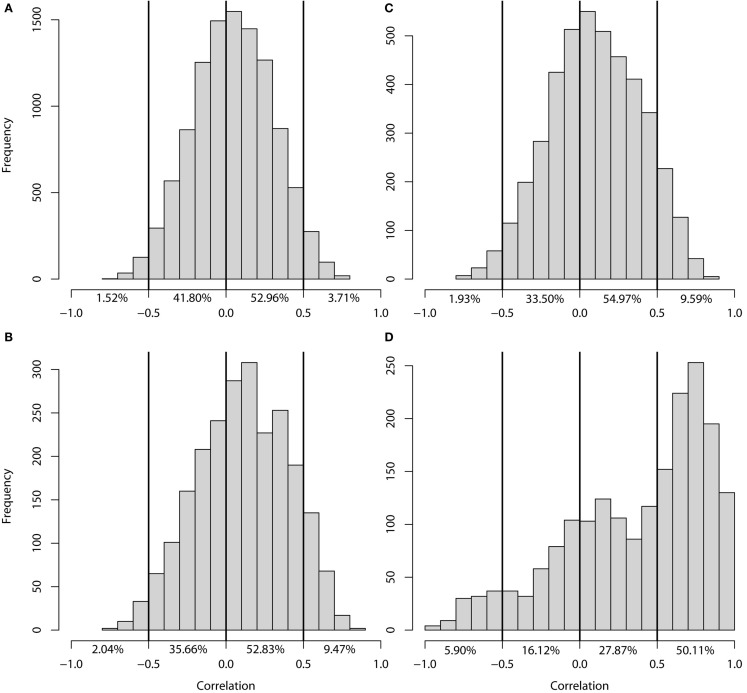
**Histogram of the correlation of −log(P) profiles per gene**. Correlation between the −log(P) profiles of genes **(A)** without eQTLs in either experiment, **(B)** with an eQTL in GG1 only, **(C)** with an eQTL in GG2 only, **(D)** with an eQTL in both experiments.

To determine the similarity in genetic regulation of each gene between the two experiments we compared – log(P) eQTL profiles. These profiles depict the significance of linkage of variation in transcript levels at every marker position along the genome, multiplied by the sign of the additive effect at each marker position. For the genes without significant eQTLs in either experiment (10693 genes) we observed that the correlation coefficients between the profiles were normally distributed around zero (Figure 3A). This was also seen for the genes with an eQTL in one experiment only (2307 genes in GG1 and4293 genes in GG2), although the distribution was slightly positively skewed (Figures 3B,C). Genes with an eQTL just below the threshold in one experiment and a significant eQTL in the other might have caused the positive skewing.

**Figure 4:**
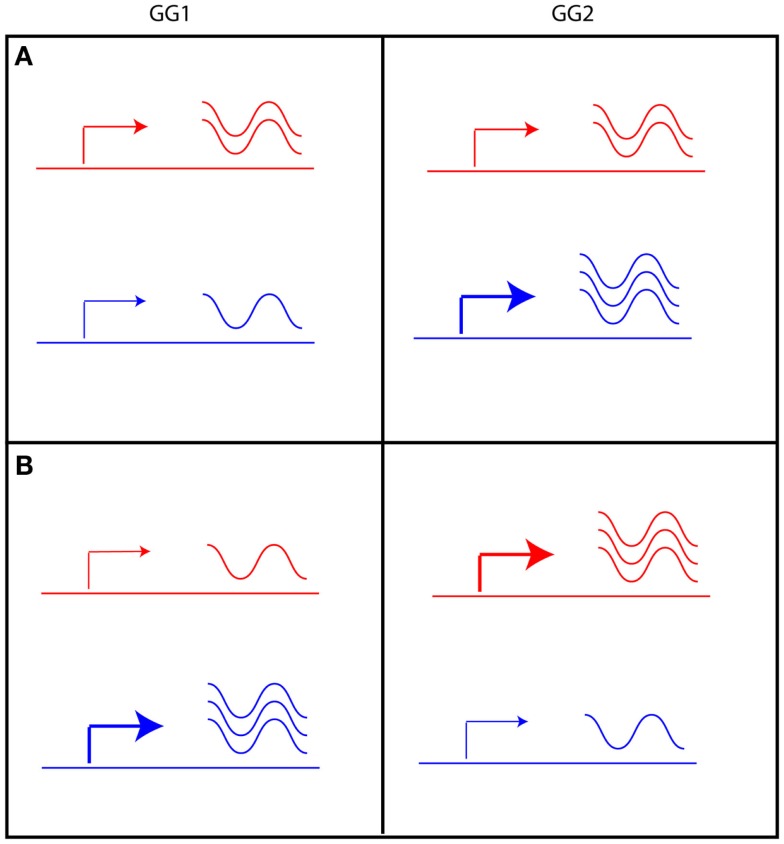
**Two scenarios can explain eQTLs with opposite effect in GG1 and GG2**. The level of expression of a given gene in L*er* (red) and Cvi (blue) is indicated by the arrow, indicating low (thin arrow) or high (thick arrow) promoter activity resulting in a low or high level of transcript (indicated by the waving lines). **(A)** gene expression in L*er* is unchanged, but in Cvi it is plastically regulated. **(B)** gene expression is plastically regulated in both L*er* and Cvi, but activation of expression is stronger in GG1 of the Cvi allele and in GG2 of the L*er* allele.

Of the genes with an eQTL in both experiments (1912), half showed a strong positive correlation (higher than 0.5; 954 genes) between their eQTL profiles (Figure 3D). The transcript levels of these genes are regulated by similar loci in the two experiments. Surprisingly, almost 6% of the genes with an eQTL in both experiments (112 genes) show a strong negative correlation (less than −0.5) between their eQTL profiles. The transcript levels of these genes are affected by the same loci in both experiments but in an opposite manner. A possible explanation is that the expression level of a gene with negatively correlated eQTL profiles could be constant in one parental line, but plastically regulated in the other (Figure 4A). Alternatively, the gene is plastically regulated in both parents, but each showing the strongest activation under different conditions, e.g., in the *Ler* parent in GG1 and in the Cvi parent in GG2 (Figure 4B).

When looking at the individual eQTLs, we can identify 135 eQTLs with opposing additive effects between the two experiments (from the total of 1129 eQTLs corresponding to 1095 genes). Gene Ontology (GO) analysis showed that these genes are enriched for the molecular function categories “glutathione transferase activity” and “lyase activity” and the biological process categories “alcohol metabolic process” and “negative regulation of cell cycle process” (Table 3).

**Table 3:** **Overrepresented GO categories in lists of genes with overlapping eQTLs in both GG2 and GG1 with opposite additive effect**.

GO category	Genes in GO cat	Genes matched in GO cat	Adjusted *p*-value	Term	Ont
GO:0004364	48	At1g27140 At1g59670 At1g59700 At1g65820	0.002533	Glutathione transferase activity	MF
GO:0016829	331	At4g37870 At2g20340 At3g54920 At3g56060 At4g27070 At4g23600 At2g20610	0.036424	Lyase activity	MF
GO:0006066	229	At5g35790 At3g56060 At4g37870 At1g17890 At4g12110 At1g20330	0.024179	Alcohol metabolic process	BP
GO:0010948	11	At1g20330 At3g18524	0.033681	Negative regulation of cell cycle process	BP

*Categories are significantly enriched with an adjusted *p*-value < 0.05. The ontology type can be Biological Process (BP) or Molecular Function (MF)*.

Among the 16 genes annotated to these enriched groups are notable examples that can be linked to the low light response. *TRYPTOPHAN SYNTHASE BETA-SUBUNIT 2* (*TSB2*At4g27070) and *SUPERROOT 1* (*SUR1* At2g20610) are involved in tryptophan metabolism and glucosinolate biosynthesis, that both impact auxin homeostasis (Bender and Celenza, 2009). Also the plastically regulated gene *STEROL METHYLTRANSFERASE 2* (*SMT2* At1g20330) that is annotated to the enriched GO categories is also located under the chromosome 1 HTR and is locally regulated. *smt2* mutants are affected in sterol composition of the membrane and in the biosynthesis of brassinosteroid hormones (BR; Chung et al., 2010). Together with auxin, this hormone is required for the SAS in response to low light (Kozuka et al., 2010; Keuskamp et al., 2011). The regulation of expression of these genes is clearly dependent on the interaction of genotype with the environment and constitutes therefore a striking example of plastic regulation.

### Promoter elements enriched in the HTR-regulated genes

The variation in transcript levels in low light treated leaves between L*er* and Cvi (GG2) is strongly determined by the HTR on the top of chromosome 1 that affects the expression of 380 genes. The promoters of these genes are enriched for the Evening Element promoter motif (hypergeometric test *p* < 10^–7^ found in 53 genes) that is found in the promoter regions of circadian-regulated genes (Harmer et al., 2000). In 31 of these 53 promoters, also the Ibox promoter motif is enriched (hypergeometric test *p* < 10^−4^) that is a conserved protein binding motif upstream of light-regulated genes (Giuliano et al., 1988). Of the 53 clock-regulated genes, 23 are responsive to low light (present in our low light-responsive gene list), including *TIMING OF CAB EXPRESSION 1* (*TOC1*, At5g61380), and *LUX ARRHYTHMO* (*LUX*, At3g46640) that are both part of the central oscillator of the clock and active in the evening loop (Nakamichi, 2011). The expression of these genes is repressed by the MYB transcription factors *CIRCADIAN CLOCK ASSOCIATED 1* (*CCA1*, At2g46830) and *LATE ELONGATED HYPOCOTYL* (*LHY* At1g01060) that bind to the evening element promoter motif (Wang and Tobin, 1998; Alabadi et al., 2001), but neither gene is located under the HTR. From the 3975 circadian-regulated genes published by Covington et al. (2008), 153 have an eQTL on the HTR, of which 49 are responsive to low light. This is a significant enrichment (hypergeometric tests *p* < 3*10^−15^ and *p* < 2*10^−10^ respectively) and suggests that a locus under the HTR is regulating part of the low light response by affecting the circadian clock. *GIGANTEA* (*GI*, At1g22770), a well known and important light-sensitive regulator of the circadian clock that is regulated at the post-translational level (Martin-Tryon et al., 2007), underlies the HTR and is differentially regulated. *GI* would therefore make a likely candidate regulator for the HTR-mediated low light responses associated with the circadian clock.

### HTR-regulated genes identified by expression profiling of a NIL

**Figure 5:**
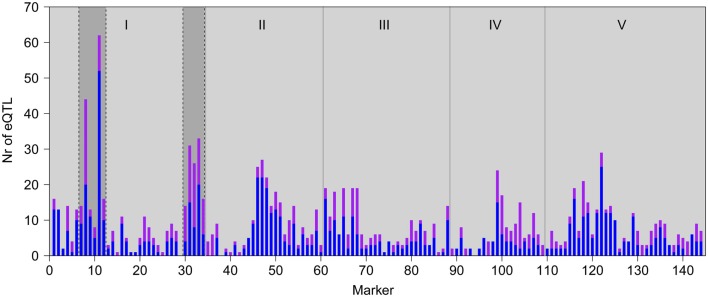
**Genomic distributions of eQTLs of the genes with differentially abundant transcripts between LCN1-10 and L*er***. Bars represent the number of local (*in cis*; purple) and distant (*in trans*; blue) eQTLs detected at each marker position. Gray vertical lines depict chromosomal borders, chromosomes (I-V) are indicated at the top. Cvi introgressions of LCN1-10 are indicated by the darker gray areas on chromosome I.

In order to further dissect the effect of the major regulatory locus on the low light transcriptional response, we determined the effect of the Cvi alleles in the HTR region on genome wide expression. To this end we used a NIL, LCN1-10 (Keurentjes et al., 2006), that has two small Cvi introgressions on chromosome 1 in the otherwise L*er* background. The first introgression ranges from position ∼5–∼8.2 Mbp (∼950 genes) and the second introgression ranges from ∼26.5–∼30.4 Mbp (∼1150 genes). The major HTR maps to the first introgression, as well as a phenotypic QTL for the SAS. In addition, line LCN1-10 confirmed this phenotypic QTL, as it is slightly larger with a less compact rosette and more upright (hyponastic) leaves than L*er* (Snoek and Peeters, personal communication). The effect of the Cvi introgressions in L*er* on gene expression was determined by comparing transcript levels of LCN1-10 to L*er* after a 3 h low light treatment. We identified 2282 differentially expressed genes (fdr < 0.05), of which 166 are physically located in the first introgression and 155 in the second. This means that the majority of differentially expressed genes (1961, ∼85%) is not physically located in the introgressions and thus must result from *trans*-regulation. eQTLs were found for 1072 (∼45%) of the 2282 LCN1-10/L*er* differential genes, of which 21% mapped to one of the introgressions, 157 on the first and 120 genes on the second introgression (Figure 5).

This is an enrichment compared to the 15% of eQTLs that map to the introgressions in the complete dataset (hypergeometric test *p* < 1*10^−9^). The eQTLs on the first introgression were mainly distant (90 eQTLs of 157; 57%), as would be expected from an HTR. In contrast, relatively more local eQTLs mapped to the second introgression (189 eQTLs of 272; 69%). The remainder of the differentially abundant transcripts with eQTLs did not map to one of the introgressions, which could be due to indirect regulatory effects.

Of the 2282 differentially abundant transcripts between LCN1-10 and L*er*, a limited number of 660 genes (20%) have differentially abundant transcripts between Cvi and L*er* as well. In this subset two genes only are regulated by the HTR and responsive to low light. At1g55960 encodes for a polyketide cyclase/dehydrase and lipid transport superfamily protein and is strongly co-expressed, in public microarray data, with the core circadian genes *CIRCADIAN CLOCK ASSOCIATED 1* (*CCA1*, At2g46830) and *LATE ELONGATED HYPOCOTYL* (*LHY*, At1g01060), implying the involvement of this gene in regulation of the clock by the HTR. The second gene, *PTO-INTERACTING 1-4* (*PTI1-4*, At2g47060), is a member of the PTI1-like serine/threonine protein kinases that interacts with *MITOGEN-ACTIVATED PROTEIN KINASE 3* (*MPK3*, At3g45640) and *MPK6* (At2g43790) and the ACG kinase *OXIDATIVE SIGNAL-INDUCIBLE1* (*OXI1/AGC2-1*, At3g25250; Forzani et al., 2011). This OXI1-MAPK cascade is activated by lipids and ROS and necessary for oxidative burst-mediated signaling (Rentel et al., 2004; Anthony et al., 2006; Forzani et al., 2011). Analysis of the differential expression between L*er* and NIL LCN1-10 allowed the extraction of candidate genes for the low light response that are robustly regulated by the HTR.

### Joint action or common regulation?

**Figure 6:**
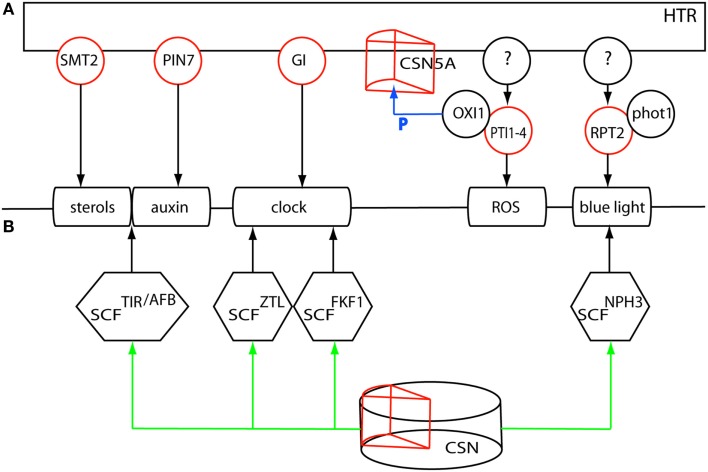
**(A)** Multiple regulatory loci underlying the HTR could affect the plastically regulated processes sterol- and auxin homeostasis, the clock, ROS and blue light perception. **(B)** A single master regulatory locus, the COP9 signalosome subunit 5A underlying the HTR could affect all processes through differential deneddylation of the corresponding CRL complexes. Red circles denote expression regulation of the gene by the HTR. P denotes phosphorylation of CSN5A by OXI1.

Several biological processes, revealed by the genetical genomics experiments described above, are affected by the identified plastically regulated genes and that are possibly linked to the SAS. These processes are auxin- and sterol homeostasis, the circadian clock and ROS signaling. Several HTR-located genes on chromosome 1 could each contribute to one of these processes and act in parallel (Figure 6A).

Auxin homeostasis could be differentially regulated by the locally regulated auxin efflux carrier *PIN-FORMED 7* (*PIN7*, At1g23080). Polar auxin transport and -localization might, in addition, be affected by *SMT2*, a gene that is also involved in sterol homeostasis (Pan et al., 2009). The large impact on the circadian clock could be mediated by the post-translational effects of *GI*. These genes, however, do not account for the effect of the HTR on ROS signaling, nor on the differential regulation of *ROOT PHOTOTROPISM 2* (*RPT2*, At2g30520), an interacting partner of the blue light photoreceptor phototropin 1 (phot1 At3g45780), which might be instrumental in the observed variation in low light perception between L*er* and Cvi. The proposed regulatory genes could act in parallel with other factors underlying the HTR that affect ROS signaling and blue light sensing and that jointly compose the HTR-mediated transcriptional response to low light.

It is tempting to speculate on an alternative scenario in which a single master regulatory gene on chromosome 1 affects all these processes (Figure 6B). A candidate integrating factor in this scenario could be the differential action of the ubiquitin/26S proteasome pathway. Protein stability and endocytosis are instrumental in the regulation of the clock, auxin-, and light signaling (Maraschin et al., 2009; Pan et al., 2009; Nakamichi, 2011; Roberts et al., 2011). All Cullin-RING type E3 ubiquitin ligase (CRL) complexes are deneddylated by the COP9 signalosome (CSN) affecting the activity of these CRL complexes (Schwechheimer and Isono, 2010). Subunit 5, required for CSN function, is encoded by two genes *CSN5A* (*AJH1* At1g22920) and *CSN5B* (*AJH2* At1g71230), of which *CSN5A* is underlying the HTR, shows local regulation of gene expression and might therefore differentially affect the CRL activities in the regulation of the different processes involved in the SAS. Overall, the integration of gene expression and eQTL data has allowed the selection of candidate genes and working models that now need to be functionally analyzed.

## Discussion

### Plastic regulation of transcript abundance in *Arabidopsis* revealed under low light

We analyzed the transcription levels in the RILs of the L*er*/Cvi population following low light treatment, and mapped eQTLs for 32% of all measured transcripts. For a large fraction of the genes (17%) recombination of the parental genotypes resulted in transgressive expression patterns. Furthermore, exposure of the lines to low light conditions revealed additional variation in gene expression. The genes that are known to be regulated by low light (Millenaar et al., 2006) were enriched in differentially expressed genes between the parents, and in the eQTLs in GG2. The induced transcriptional responses associated with the SAS revealed the corresponding underlying natural variation in gene regulation. Also in yeast it was observed that the induction of gene expression by heat-shock treatment revealed new sources of genetic variation specifically for the regulation of essential genes (Eng et al., 2010). Our study is the first genetical genomics analysis in *Arabidopsis* that compares two very different experimental conditions. The dynamic response to low light treatment at the transcriptional level was compared to the “steady state” expression analysis in the same population in a previous study (Keurentjes et al., 2007). The majority of eQTLs identified in both experiments (91%) were specific to each of the experiments. Distant regulation was most affected in the different experiments, as was found in genetical genomics experiments on *C. elegans* and yeast (Li et al., 2006; Smith and Kruglyak, 2008). Only a moderate number of eQTLs (9%) that are predominantly locally regulated were mapped in both our *Arabidopsis* experiments, suggesting genotype-specific regulation. Local regulation was correlated to the distribution of SNP containing genes, which is also found in a genome wide association study in *Arabidopsis* (Zhang et al., 2011), where local regulation is enriched in *cis*-acting polymorphisms. Surprisingly, several eQTLs that mapped in both experiments show opposite additive effects, a striking example of plastic regulation. The opposite effect of eQTLs in different environments has also been observed in other organisms. In yeast grown on different nutrients this phenomenon affected mainly *trans*-eQTLs (Smith and Kruglyak, 2008). In a study where *cis*-eQTLs were analyzed in human samples obtained from different tissues eQTLs were identified with “opposing allelic direction,” prompting the authors to highlight the importance of using disease-related tissues to correctly characterize the effects of disease-associated variants (Fu et al., 2012). The plastic regulation of this type in the L*er*/Cvi RIL population concerns genes that are known to be related to auxin- and sterol homeostasis, both involved in the response to low light (Keller et al., 2011; Keuskamp et al., 2011).

### Dissecting the HTR-mediated SAS

The dynamic nature of the distant eQTLs is best reflected in the presence of HTRs, which are quite distinct for each experiment. ERECTA on chromosome 2 regulated the expression of 176 genes in GG1. The main HTR in GG2, at the top of chromosome 1, regulated the expression of 380 genes, enriched in circadian and light-regulated genes. Involvement of the circadian clock in the SAS was previously shown by the identification of the evening-expressed gene *EARLY FLOWERING 3 (ELF3* At2g25930) as the causal gene for a shade avoidance QTL in the Bay × Sha RIL population linking light input into the clock (Jimenez-Gomez et al., 2010). *ELF3* was found to modulate the biological activity of *GIGANTEA* (*GI*, At1g22770) by direct binding to GI affecting its protein stability (Yu et al., 2008). Developmental growth is gated by the clock through the action of the growth promoting transcription factors PIF4 and PIF5. Where light negatively affects their protein abundance, the clock regulates their expression, reducing expression in the early night (Salter et al., 2003; Nozue et al., 2007). This balancing of transcription and protein levels by the combined action of light and the clock restricts growth to the late stages of the night. It is possible that the hyponastic growth response in the SAS is gated in a similar fashion by the clock.

We used the transcriptional response of the NIL LCN1-10 to study the effects of the Cvi alleles in the HTR in an otherwise isogenic genetic background. The differentially expressed genes between L*er* and LCN1-10 are mainly regulated in *trans*, with an enrichment for genes with an eQTL on the Cvi introgressions. This confirms the eQTLs mapped for those genes. An important result from the expression analysis of LCN1-10 is the confirmation of the direct differential regulation by the HTR of two low light-responsive genes; At1g55960, encoding a polyketide cyclase/dehydrase that might be involved in regulation of the clock, as deduced from co-expression data, and *PTI1-4*involved in ROS signaling through the OXI1-MAPK cascade. Growth, as in tip-growing root hairs, is accompanied by ROS production and signaling (Sauer et al., 2001; Foreman et al., 2003; Carol and Dolan, 2006). Also in expanding cells and growth in response to changing light conditions ROS signaling may be expected (Carol and Dolan, 2006). The ACG kinase OXI1 was found to target not only CSN5A for phosphorylation (Howden et al., 2010), but also 3′phosphoinositide-dependent kinase-1 (PDK1) a conserved lipid-activated master kinase (Anthony et al., 2004; Zalejski and Bögre, 2010). *OXI1* in turn is activated by *PDK1* that in addition regulates a number of other AGC kinases. Among these kinases is *PINOID* (*PID*At2g34650) affecting PIN localization and polar auxin transport. ROS were shown to be generated by auxin (Joo et al., 2005) and interact with auxin in adaptation to environmental stress (Tognetti et al., 2012). Genes in the pathways of tryptophan-, auxin-, and glucosinolate biosynthesis and components in auxin signaling are differentially expressed between L*er* and LCN1-10 and/or Cvi, although not many components are directly regulated by the HTR, only *PIN7* and the auxin biosynthesis genes *NIT1*(At3g44310) and *ATAMI1*(At1g08980). This might be due to the indirect effects of the differential regulation of the OXI1-MAPK cascade on PID via PDK1. PID acts at the protein level on the PINs to target them to the membrane, whereas the resulting auxin gradient affects auxin signaling and tropic growth responses (Esmon et al., 2006). When auxin also affects ROS signaling, feeding back into the ROS-activated OXI1-MAPK cascade, this would constitute transcriptional means to dampen or enhance the response to environmental stress, in this case low light.

### A candidate pleiotropic master regulator

Several processes have emerged from our analyses that suggest they are plastically regulated in response to low light. The HTR as master regulatory locus could affect these processes, through the effect of multiple polymorphic genes acting in parallel. Local regulation of gene expression points to candidate regulators for the effect of the HTR on the plastically regulated auxin- and sterol homeostasis (*PIN7* and*SMT2*) and for the differential regulation of the biological clock (GI). It is very well possible that a suite of nearby positioned *cis*-regulatory eQTLs act in concert in response to the low light treatment. In mouse, Fraser et al. (2011) have shown that adaptation of gene expression is widespread and that this adaptation can involve selection on multiple functionally related *cis*-regulated genes.

A common feature of the different processes is the ubiquitin/26S proteasome pathway linked to protein endocytosis or degradation. Ubiquitination is however executed by different types of E3 ubiquitin ligase complexes, composed of different RING-finger Cullins (CUL1, CUL2, CUL3a/b, or CUL4) bound to a wide range of substrate specifying components like the F-box proteins in the SCF complexes or the BTB/POZ proteins bound to the CUL3 scaffold proteins. All these complexes themselves, however, are also prone to degradation. Addition of RUB (RELATED TO UBIQUITIN or NEDD8), an ubiquitin homolog, activates the complex (Schwechheimer and Isono, 2010), whereas removal of RUB (deneddylation) could stabilize the complex (Stuttmann et al., 2009). Deneddylation is executed by the CSN5 subunit of the COP9 signalosome (CSN) and is required for a number of pathways, including light and phytohormone signaling (Moon et al., 2007; Schwechheimer and Isono, 2010). *CSN5A*, part of the COP9 subunit 5 and underlying the HTR, was not analyzed in GG2, but in GG1 the gene shows local regulation of expression. Mutant phenotypes of this gene have the *constitutively photomorphogenic/de-etiolated/fusca* (*cop/det/fus*) mutant phenotype (Dohmann et al., 2005) of light-grown seedlings when grown in the dark. In an analysis of targets of OXI1 CSN5A was found to be phosphorylated (Howden et al., 2010). It could very well be that the differential expression and activity of this gene between L*er* and Cvi could account for the diverse processes differentially affected by the HTR in response to low light.

Examples in the literature of COP9-regulated proteins that emerged in our study include photoreceptors and auxin-related proteins. The blue light photoreceptor PHOT1 is endocytosed or degraded dependent on ubiquitination status (Roberts et al., 2011). PHOT1 is the primary receptor under low light intensities and interacts with *NON-PHOTOTROPHIC HYPOCOTYL 3* (*NPH3* At5g64330) and *RPT2* both containing BTB/POZ protein-protein interaction domains (Sakai et al., 2000)*. RPT2* expression is light induced (Sakai et al., 2000) and regulated by the HTR. In both LCN1-10 and Cvi the differential expression of *RPT2* could reflect a differential effect in low light perception compared to L*er*.

GI affects protein stability by light-mediated interaction with members of the ZTL family of photoreceptors comprising *ZTL* (At5g57360), *FLAVIN-BINDING, KELCH REPEAT, F-BOX 1* (*FKF1* At1g68050), and *LOV KELCH PROTEIN 2* (*LKP2* At2g18915; Demarsy and Fankhauser, 2009). This interaction stabilizes ZTL and prevents the Skp-Cullin-F-box (SCF) SCF^ZTL^ mediated degradation of TOC1. The binding of GI to FKF1 however, enables the degradation of CYCLING DOF FACTOR 1 (CDF1 At5g62430), releasing *CONSTANS* (*CO* At5g15840) from its repression. *CDF1* and *FKF1* are both differentially regulated by the HTR, just like *CONSTANS-LIKE 1* (*COL1* At5g15850) and *COL2* (At3g02380).

*SMT2* could affect correct sterol composition, required for the auxin-regulated endocytocis and membrane localization of the PIN auxin transporters (Pan et al., 2009), which might also involve PIN7. Especially as the *pin7* mutant shows an impaired low light response (Keuskamp et al., 2011). Also the regulation of the transcriptional response to auxin requires SCF^TIR^-mediated degradation of the AUX/IAAs in relieving the ARF transcription factors from their repression.

Further analysis should shed more light on the proposed candidate regulator in the HTR- mediated response to low light. At the moment it is not known if ROS signaling is also influenced by for instance differential degradation or endocytosis of PTI1-4. The differential expression of *PTI1-4* does suggest regulation at the level of protein stability, like the transcript levels of *RPT2*, *GI*, *FKF1*, and *CDF1* are affected. Also the role of sterol homeostasis mediated by *SMT2* could be working in parallel to the effects of the signalosome on protein trafficking, or be an integral, downstream component. A single pleiotropic regulator does fit nicely the observed phenotypic buffering described by Fu et al. (2009). They observed that not all QTLs responsible for variation in transcript levels affect phenotypic traits. Instead they identified six major HTRs in their analysis of more than 40000 molecular traits (transcript-, metabolite-, and protein levels) and 139 phenotypes in the same L*er*/Cvi population, whose effect is not only observed at the molecular but also at the phenotypic level, as opposed to many eQTLs that do not affect phenotypes in a robust system (Bergman and Siegal, 2003). They do show, however, eQTLs and protein level QTLs co-locating at the position of the HTR identified in GG2.

In conclusion, we find that natural variation in gene expression regulation is not only strongly influenced by new combinations of *Ler* and Cvi alleles (giving transgression) but also to a large extent by the experimental conditions (environment and developmental stage). In particular the distantly or *trans*-regulated genes show most interaction with the environment. Although the small numbers of constitutive eQTLs are predominantly locally regulated, their effect does not solely depend on genotype, but can be plastically regulated as well. Based on the plastic regulation in response to low light we were able to dissect the major effect of the HTR on transcription and we propose *CSN5A* as a candidate regulatory factor to underlie this locus and to differentially affect transcript- and possibly also protein levels in response to low light.

## Materials and Methods

### Plant growth

To identify natural variation in transcript abundance, 120 RILs of the L*er*xCvi population (Alonso-Blanco et al., 1998) were grown in one batch, divided over 10 trays containing three plants of 14 individual genotypes, parents included. In this way we obtained three plants per RIL and nine per parent. Plants were grown under short days (9 h light, 15 dark), a light intensity of 200 μmol m^−2^ s^−1^ and watered three times per week.

### Shade treatment and harvest of material

All RILs and parents were treated with 3 h of neutral shade (ca 10% of growth conditions) 21 days after transfer to soil (24 days after germination). The three most responsive leaves (petiole and lamina) per plant were harvested and pooled per genotype for RNA isolation and transcription profiling.

### Microarray analysis

All procedures were described in de Jong et al. (2006). In short, RNA was extracted and purified using the RNeasy kit (Qiagen, Valencia, USA). Amplification and labeling was performed with the Message Amp aRNA kit (Ambion, Austin, USA). Amplified RNA was used to generate labeled cDNA with incorporation of 5-(3-aminoallyl)-dUTP and labeled with either Cy3 or Cy5 mono-reactive dye (Amersham, Piscataway, USA). All cDNA products were purified using the QIAquick PCR purification Kit (Qiagen, Valencia, USA). The CATMA (Allemeersch et al., 2005) *Arabidopsis* DNA microarrays were provided by Utrecht University (the Netherlands) and were produced from a set of 150-450-mers representing 20833 unique genes. Arrays were scanned using ScanArray Express HT (PerkinElmer, Wellesley, USA) and quantified with Imagene 6.0 (BioDiscovery, El Segundo, USA).

The Limma package (Smyth and Speed, 2003) for the statistical work environment R was used for normalization. All array data are submitted to Array Express accession number E-MTAB-1357.

### Statistical analysis

To determine differential abundance of transcripts between the two parents, we applied a linear model using the Limma package for the statistical work environment R (Smyth, 2004, 2005). For each gene the *p-*value and the corresponding *q* values (Storey and Tibshirani, 2003) were computed.

The procedures developed in GG1 were used to calculate the broad sense heritability (H^2^) in the parents and RILs. Transgressive segregation was determined in terms of the standard deviation of the individual parents (Brem and Kruglyak, 2005). We calculated the number of RILs (*n* = 20), whose expression level lay above μ_max_ + 2*SD_max_ or below μ_min_ − 2*SD_min_; where μ and SD are the mean and the standard deviation of the parental phenotypic values, respectively, and max indicates the parent with the highest value and min indicates the parent with the lowest value.

### Multiple QTL analysis

eQTLs were mapped using the procedures based on MQM mapping, as developed in GG1. A genome wide *p*-value threshold of 2.23 × 10^−3^ at α = 0.05 for a single trait was estimated by a 10,000 permutation test (Churchill and Doerge, 1994). But for a study with 24,065 gene transcripts, we controlled the false discovery rate (FDR) based on the pool of *p*-values for all markers and all transcripts. Because the *p*-values are correlated when markers are linked, the FDR increases depending on the number of markers on a chromosome (Benjamini and Yekutieli, 2005). In our experiment the maximum number of markers reached 35 (chromosome 5) and a simulation analysis (not shown) using Storey’s algorithm to control the FDR (Storey, 2002) at a desired level indeed showed a 4.4-fold increase of the actual FDR. To account for this we corrected the FDR by a factor 5 and calculated the genome wide *p*-value threshold at Storey’s FDR of 0.01 for all gene-marker *p*-values to make sure that the real FDR rate is below 0.05 (corrected FDR = 0.05). The estimated *p*-value threshold then corresponded to 5.29 × 10^−5^. As neighboring markers stand for partially/largely similar tests, we decided to leave them out, resulting in significance thresholds of −log(P) = 3.83 in GG1 and 3.62 in GG2. These threshold levels were used as significance thresholds for the detection of eQTLs.

### Correlation of SNP frequency, gene density, and eQTL distribution

The SNP set published in (Nordborg et al., 2005) was used. The SNP frequency and gene density were determined for each marker by counting the number of SNPs or genes between the flanking markers of the marker under study (marker locus). Indels were treated as a single polymorphism and the number of SNPs was corrected for the number of sampled sequences per marker locus. The number of eQTL at the marker locus, were determined by the number of eQTL above the established genome wide threshold. The Pearson correlation between numbers of SNPs and eQTLs was calculated and significance of correlation was determined using a *t*-test.

### Additional databases and tools used

We used the Athena tool (O’Connor et al., 2005) to extract enriched transcription factor binding sites in the genes regulated by the HTR.

Overrepresentation of Gene Ontology categories was analyzed with the R package GOstats (Falcon and Gentleman, 2007).

Hypergeometric tests were applied to assess significance of overrepresentation either incorporated in the Athena tool or from the GOstats or Hypergeometric R package.

Co-expression data, based on publicly available microarray data, was obtained from the *Arabidopsis thaliana trans*-factor and *cis*-element prediction database ATTED-II (Obayashi et al., 2007).

## Conflict of Interest Statement

The authors declare that the research was conducted in the absence of any commercial or financial relationships that could be construed as a potential conflict of interest.
